# Co-infection with *Schistosoma haematobium* modulates the gene expression profile of malaria infection in schoolchildren in Gabon

**DOI:** 10.1186/1475-2875-13-S1-P30

**Published:** 2014-09-22

**Authors:** Katie Ewer, Julius Muller, Marielle Haks, Marguerite Massinga Loembe, Maria Yazdanbakhsh, Magali Matsumiya, Edwin Quinten, Tom Ottenhoff, Peter Kremsner, Adrian Hill, Hermelijn Smits, Akim Adegnika

**Affiliations:** 1 The Jenner Institute, University of Oxford, Oxford, UK; 2 Leiden University Medical Centre, Leiden, The Netherlands; 3 Centre de Recherches Médicale de Lambaréné, Fondation Internationale de l’Hôpital Albert Schweitzer, Lamarene, Gabon; 4 Institut für Tropenmedizin, Tübingen, Germany

## Background

Helminths and malaria parasites inhabit the same geo graphical areas and coinfection is frequently observed. The immunological interaction between helminth and malaria infections is highly relevant to disease control and elimination, yet is poorly understood and understudied. Chronic helminth infection causes immune hyporesponsiveness and stimulates anti-inflammatory cytokine production, which in turn modulates the host immune response to malaria. In epidemiological studies, many data are conflicting with some reporting protective effects against malaria for *Ascaris lumbricoides* and *Schistosoma haematobium* while infection with hookworm or *S. mansoni* may increase the incidence of malaria and worsen pathology.

## Materials and methods

We undertook a prospective study of schoolchildren aged 5-16 in an area endemic for schistosomiasis near Lambaréné in Gabon. 425 children were recruited and demographic data was collected. Laboratory analysis for *P. falciparum* malaria infection (thick blood smear and rapid test) and *S. haematobium* infection (urine microscopy) was performed and treatment was provided to infected children. Children were followed up locally for malaria-like symptoms, actively for two months and passively for a further 2 years. At recruitment, whole blood was collected into Paxgene tubes from a subset of children for transcriptional profiling among uninfected (n = 12), *S. haematobium* infected (n = 16), malaria infected (asymptomatic, n = 8) or coinfected (n = 13) children. RNA was extracted, amplified and hybridized to Illumina HT12v4 microarrays.

## Results

In the full cohort age, gender and schistosomiasis were not associated with malaria and malaria incidence among these schoolchildren was 0.4 episodes per person per year. Analysis of the microarray data revealed monoinfection with either *S. haematobium* or malaria resulted in relatively insignificant changes to the transcriptome relative to healthy controls at the 10% false discovery level. There was also no difference in gene expression profile between healthy controls and coinfected children. However, between malaria infected and coinfected children there were 240 differentially expressed genes with 180 genes upregulated and 60 downregulated in the coinfected group. Specific genes of relevance to malaria infection, including CD8a, were significantly downregulated in malaria-infected compared to coinfected children. Pathway analysis revealed substantial modulation of gene expression in defence and immune responses and in particular modulation of genes in the Fcγ-receptor-mediated phagocytosis pathway.

## Conclusions

Confirmatory experiments are underway to confirm these intriguing insights into the immunomodulatory effect of *S. haematobium* on *P. falciparum* malaria in a key population for public health intervention.

